# Infant mortality among the Canadian-born offspring of immigrants and non-immigrants in Canada: a population-based study

**DOI:** 10.1186/s12963-016-0101-5

**Published:** 2016-08-30

**Authors:** Zoua M. Vang

**Affiliations:** Sociology Department, McGill University, 855 Sherbrooke Street West, Montreal, QC H3A 2T7 Canada

**Keywords:** Healthy immigrant effect, Infant mortality, Maternal child health, Canada, Population-based data

## Abstract

**Background:**

Adult immigrants in Canada have a survival advantage over their Canadian-born counterparts. It is unknown whether migrants are able to transmit their survival advantage to their Canadian-born children.

**Methods:**

Neonatal and postneonatal mortality between the Canadian-born population and 12 immigrant subgroups were compared using 1990–2005 linked birth-infant death records. Age-at-death specific mortality rates and rate differences were calculated by nativity status and maternal birthplace. A chi-square statistic was used to compare group differences in maternal sociodemographic characteristics. Multivariate survival analysis was used to estimate the effect of maternal birthplace on neonatal and postneonatal mortality, net of maternal sociodemographic and infant characteristics.

**Results:**

Overall, immigrants had lower rates of neonatal and postneonatal mortality than the Canadian-born population. But the adjusted risk of neonatal mortality was higher for Sub-Saharan African (hazard ratio [HR] = 1.32; 95 % confidence interval [CI] = 1.05, 1.66), Haitian (HR = 2.29, 95 % CI = 1.90, 2.76), non-Spanish Caribbean (HR = 1.38; 95 % CI = 1.01, 1.89), and Pakistani (HR = 1.87; 95 % CI = 1.31, 2.68) migrants relative to Canadian-born women. There were fewer significant disparities in postneonatal death, with higher adjusted risks of mortality observed for Pakistani (HR = 2.67, 95 % CI = 1.77, 4.02) and Haitian (HR = 1.41, 95 % CI = 1.02, 1.97) migrants only.

**Conclusion:**

Inequalities in infant mortality are more concentrated in the neonatal period. Contingent on surviving the first 27 days after birth, the infants of most immigrants (except those from Haiti and Pakistan) have the same chances of survival as the infants of Canadian-born women. Improvements in prenatal care and access to postpartum care may reduce disparities in infant mortality.

## Background

During the 1960s, 27 out of every 1000 live-born infants in Canada died before reaching their first birthday [1]. By 1990, the infant mortality rate (IMR; deaths per 1000 live births) dropped to 6.5 [2] and declined even further to 5.0 in 2007 [3]. Despite improvements in infant survival in Canada, substantial variation in infant mortality within its diverse population remains [4]. For example, 1991–2000 infant mortality rates for Inuit and First Nations people in Québec were four and two times higher than that observed for the non-Aboriginal population, respectively [5].^1^ An important characteristic that may stratify infants into different risk groups is mother’s nativity status and, among immigrants, maternal country/region of origin (hereafter maternal birthplace). Yet, research to date has not investigated infant mortality among the offspring of immigrants relative to the children of Canadian-born women. The paucity of research is surprising given that approximately 22 % of all births annually in Canada are to immigrant women [6].

Research on infant mortality in the United States (US) has consistently documented a survival advantage among the US-born offspring of immigrant women [7–9]. This survival advantage is part of a larger phenomenon known as the healthy immigrant effect, whereby international migrants are healthier than the native-born population in the receiving country [10]. Migrant selection processes at both the individual and state level are considered to be the underlying mechanisms behind migrants’ superior health [11]. Positive health selection of migrants into a receiving country may occur because of immigration policies that directly or indirectly favor healthy individuals. For example, Canada’s point system rewards migrants with host language skills, higher education, work experience, and other characteristics that will contribute to their success in the Canadian labor market [12]. These same individuals are more likely to be healthy because more educated and economically successful people command more resources which enable them to better manage health and illness [13]. Migrants’ superior health may also be the result of self-selection processes wherein individuals who are robust enough to endure the journey are more likely to uproot [11]. The healthy immigrant effect has been documented for mortality among *adults* in Canada [14–16]. However, it remains to be seen whether or not migrants are able to transmit their survival advantage to their Canadian-born children.

The overall objective of this study is to compare neonatal and postneonatal mortality between the Canadian-born population and immigrants from diverse origin countries/regions using1990-2005 linked birth-infant death records. Given the healthy immigrant effect observed for adult migrants, the hypothesis is that infant mortality will be lower among the offspring of immigrants than the children of Canadian-born women. The influence of maternal sociodemographic characteristics on the association between maternal birthplace and neonatal/postneonatal mortality is also evaluated. This paper contributes to the growing literature on migrant health in Canada by showing that the Canadian-born children of immigrants also have a survival advantage. However, migrant offspring’s lower mortality risk varies across immigrant subgroups. The children of European, East/Southeast Asian, and Latin American migrants have a clear survival advantage relative to the children of Canadian-born women, especially in the neonatal period. However, there are exceptions to this healthy immigrant effect, with the children of Haitian and Pakistani migrants being particularly vulnerable to death throughout infancy.

## Methods

### Data source and study population

Data are derived from the Canadian linked live birth-infant death file created by Statistics Canada through probabilistic linkage of birth and death registrations [17]. Details about the data linkage, including validity, have been published elsewhere [18]. The data consisted of 3,370,641 singleton live births occurring during the years 1990–2005 followed through the first year of life and linked with 14,411 deaths that occurred prior to the infant’s first birthday. Access to the data was provided by Statistics Canada through a Research Data Centre (RDC) Pilot Project. Data were pooled across 16 years to enable analysis of infant mortality by nativity status over time and to produce sufficient sample sizes to assess variation in infant death across different immigrant subgroups.

Births to women residing in Ontario were excluded because of relatively large proportions of unlinked deaths in some birth cohorts, lack of universal birth certification, and poor data quality [17]. This strategy of omitting Ontario data from national estimates of infant mortality is consistent with prior research [3, 17, 19, 20]. Approximately 39 % of all births to women residing in Canada during the study period occurred in Ontario and 29 % of the Ontario births were to immigrant women (author’s own calculations based on the 1990–2005 linked birth-death records for all provinces and territories). The exclusion limits generalization of the findings to all of Canada, especially since one-third of the total population resides in Ontario and the province is a major destination for immigrants [21].

Births with missing information on mother’s country of birth (*N* = 74,532; 2 %), maternal age (*N* = 324; <1 %), and infant sex (*N* = 179; <1 %) were omitted from the analysis. Consistent with prior research [3, 18, 20, 22], births with weights under 500 g (*N* = 2228; <1 %) and less than 22 completed weeks of gestation (*N* = 178; <1 %) were deleted because survival of infants born before 22 weeks gestation or under 500 grams is rare [23]; thus, exclusion of these births that are at the threshold of viability produces more conservative infant mortality rates that are comparable with national estimates published by the Public Health Agency of Canada [3, 18].

### Variables

The dependent variables are neonatal (0 to 27 days) and postneonatal (28 to 364 days) mortality. Age-at-death specific mortality rates were calculated because of different etiologies. Neonatal mortality is more strongly associated with the mother’s biological endowment and complications during pregnancy and childbirth [7, 24, 25]. In contrast, exogenous environmental factors and maternal behavior play a more prominent role in postneonatal mortality because death during this period is more often associated with infectious diseases and accidents [26].

Information on mother’s country of birth, available on the birth certificate, was used to distinguish Canadian-born women (reference group) from foreign-born women and to further categorize the immigrants into 12 country/region of origin categories: US, North Africa, Sub-Saharan Africa, Haiti, the non-Spanish Caribbean (excluding Haiti)^2^, Latin America, Pakistan, South Asia (excluding Pakistan), West/Central Asia, East/Southeast Asia, Europe, and the rest of the world. These categories were determined based on United Nations 2013 world region classification [27], similarities in origin cultures, and preliminary comparison of IMRs among select individual countries within each region for internal consistency (see Appendix for further details).

Group variation in infant and maternal characteristics may account for maternal birthplace differences in infant mortality. Therefore, adjustment for known determinants of infant mortality was made in multivariate analyses: infant sex (male, female), maternal age (<20, 20–24, 25–29, 30–34, 35 and older), parity (primiparous, multiparous and missing), and marital status (married, single, and other/missing). The reference categories for these covariates are female, 20–24, multiparous, and married, respectively. Infant mortality rates tend to be higher for boys than girls because of sex differences in genetics and biological endowments, making boys more vulnerable to disease [28]. Both very young and very old maternal age is associated with greater risk of infant death owing to more complications during pregnancy and childbirth [29, 30]. First births are associated with increased infant mortality because first-time mothers may be less experienced at child care and may have fewer resources to offer children compared to experienced mothers with older children [31]. Married status is associated with lower infant mortality because of a presumed protective environment for childbearing due to greater economic and social resources [32, 33]. Birth cohort is included as a control variable because infant mortality in Canada has shifted over time [2, 22]. Birth cohorts are disaggregated into four categories: 1990–1993 (reference), 1993–1997, 1998–2001, and 2002–2005. Prior research has documented important regional variation in infant mortality in Canada [34]. Province was included in all models as a fixed effect to control for any unaccounted regional effects. The categories for province are Québec (reference), British Columbia, Prairies (Alberta, Manitoba, Saskatchewan, and Winnipeg), and Other (Atlantic provinces, Northwest Territory, Yukon, Nunavut). Finally, Québec is the only province that collects complete information on mother’s education on the birth certificate, thereby permitting examination of education as a confounder in multivariate analysis (for Québec subsample only). Maternal education is a categorical variable corresponding to less than a high school education, high school certificate or equivalent degree, some college (including CEGEP^3^), and bachelor degree or higher. Observations with missing information on mother’s education (*N* = 118,475 or 11 % of the Québec analytical sample) were included as a fifth “missing” category.

### Analytical strategy

**Fig. 1:**
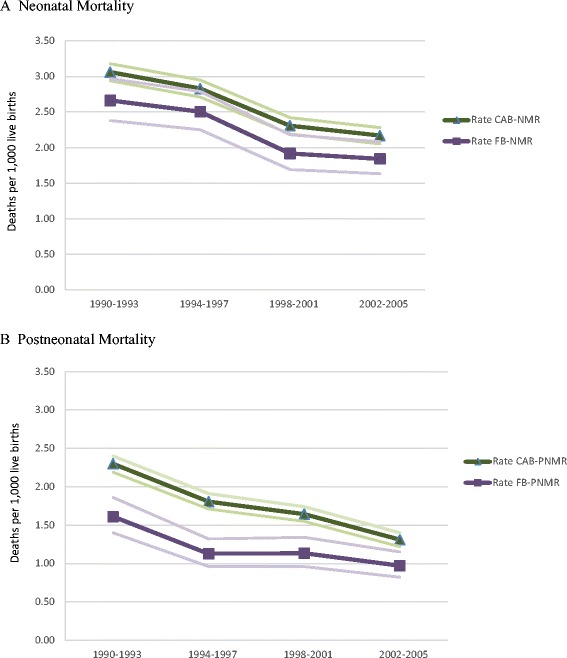
Crude neonatal and postneonatal mortality rates and 95 % confidence intervals for Canadian-born and foreign-born women by birth cohort: Canada (excluding Ontario), 1990–2005. Legend: CAB = Canadian-born; FB = Foreign-born; NMR = neonatal mortality rate; PNMR = postneonatal mortality rate

Group differences in the distribution of maternal and infant characteristics were compared using χ^2^ test statistics. Crude neonatal (NMR) and postneonatal (PNMR) mortality rates and 95 % confidence intervals (CI) were calculated for Canadian-born and foreign-born women and a nativity gap was generated for each birth cohort (Fig. 1). The nativity gap is calculated as the difference between the Canadian-born NMR/PNMR and the foreign-born NMR/PNMR. Additional NMR, PNMR, and 95 % CIs were calculated by maternal birthplace and absolute and relative rate differences were computed to enable comparisons between each of the immigrant subgroups and the Canadian-born population (Table 2). All mortality rates were calculated using live births in the denominator (as opposed to infants at risk).

Multivariate survival analysis was used to examine the association between maternal birthplace and infant death, net of maternal sociodemographic characteristics. In preliminary analysis, the proportionality assumption required for the Cox hazards model – the most popular regression method for analysis of time-to-event data [35] – was tested with Schoenfeld residuals and graphically examined with log (-log Survival) curves, for neonatal and postneonatal deaths separately [36]. These diagnostic tests revealed that the proportionality assumption applied to postneonatal but not neonatal deaths; hence, different survival analysis regression methods were used to estimate mortality risk in the neonatal and postneonatal periods. Weibull regression in the proportional hazard metric was used to estimate the association between maternal birthplace and neonatal mortality. The Weibull model is expressed as

1 $$ h\left(t\Big|{x}_j\right)=p{t}^{p-1} \exp \left({\beta}_0+{x}_j{\beta}_x\right) $$

where *p*, indicates the shape of the hazard function. For infant mortality, we would expect to see values of *p* < 1, indicating a decreasing failure rate over time [36]. The Cox proportional hazards model was used to estimate the association between maternal birthplace and postneonatal mortality. The Cox model is defined as:

2 $$ h\left(t\Big|{x}_j\right)={h}_0(t) \exp \left({\beta}_0+{x}_j{\beta}_x\right) $$

where ${h}_0$ is the baseline hazard. For both Eqs. 1 and 2, ${x}_j$ is a vector of covariates with corresponding parameters $\beta$ and *t* is time. The *stcox* and *streg* procedures in Stata 14 were used to produce hazard ratios (HR) and corresponding 95 % CIs [37].

The multivariate analysis was performed for all infants without stratification as well as separately for male and female infants. The stratified analysis investigated infant sex as a potential effect modifier of the association between maternal birthplace and neonatal/postneonatal mortality. As noted above, boys are more susceptible to infant mortality because of biological sex differences in disease vulnerability. However, sociocultural factors such as son preference may elevate infant mortality for girls relative to boys in some immigrant subgroups. For example, China and India are two immigrant-sending countries where son preference has been well-documented [38, 39]. If son preference remains strong among these immigrants in Canada then this practice might translate into differential investment in boys and girls, which may in turn elevate the risk of female infant mortality. There is some evidence of continued son preference among some East and South Asian immigrant subgroups in Canada [40–42].

Finally, additional multivariate analysis was performed on a subsample of women in Québec. The subanalysis sample was restricted to Canadian-born women and Haitian immigrants because the sociodemographic composition of the overall immigrant population in Québec is different from the rest of Canada [21]. Consequently, comparison of results for immigrants in Québec and the rest of Canada may be misleading. However, because 99 % of Haitian immigrants in Canada reside in Québec, some generalizations can be made based on Québec data. Weibull and Cox regressions were used to estimate the association between maternal birthplace (Haiti versus Canada) and neonatal and postneonatal mortality, respectively, net of mother’s education and other covariates noted above.

## Results

### Descriptive results

Maternal sociodemographic characteristics by mother’s birthplace are shown in Table 1. Immigrant women had somewhat lower risk profiles than Canadian-born women. For instance, immigrants (76–98 %) were more likely to be married than Canadian-born women (57 %), except for Haitian (50 %) and non-Spanish Caribbean migrants (64 %). Delivery at a very young age (<20 years) was less likely in immigrant (1–6 %) than Canadian-born women (7 %). Immigrants also tend to be more experienced mothers, with higher proportions of multiparous mothers among migrants from the US (62 %), Sub-Saharan Africa (61 %), Haiti (63 %), non-Spanish Caribbean (61 %), Latin America (62 %), Pakistan (66 %), and Central/West Asia (65 %) than Canadian-born women (55 %). These favorable characteristics may explain why immigrant offspring have lower mortality than Canadian-born offspring and thus must be taken into account in the multivariate analysis.

**Table 1:** Characteristics of mothers and infants for singleton live births by maternal birthplace: Canada (excluding Ontario), 1990–2005

		Foreign-Born by Mother’s Country/Region of Birth											
	Canadian-born	US	North Africa	Sub-Saharan Africa	Haiti	Non-Spanish Caribbean	Latin America	Pakistan	South Asia	Central/West Asia	East/SE Asia	Europe	Rest of the World
Infant sex, %													
Male	51.3	51.3	51.6	50.7	50.1	50.4	51.5	51.2	52.7	51.2	51.7	51.5	50.9
Female (reference)	48.7	48.7	48.4	49.3	49.9	49.6	48.5	48.8	47.3	48.8	48.4	48.5	49.1
Mother's Age, %													
Under 20	7.0	3.6	0.9	1.9	3.7	3.9	5.5	1.2	0.9	3.5	1.1	1.5	1.8
20–24 (reference)	21.6	16.5	10.8	13.1	15.7	16.6	21.2	19.9	22.4	21.1	8.1	11.0	17.4
25–29	34.1	30.6	29.8	32.2	27.9	29.4	31.1	37.7	40.3	33.9	27.0	31.0	34.2
30–34	26.6	30.5	35.1	35.0	30.0	30.1	27.0	27.6	26.1	27.7	38.1	35.5	31.3
35+	10.7	18.8	23.5	17.9	22.8	20.0	15.2	13.6	10.4	13.8	25.7	21.0	15.3
Marital status, %													
Single	37.7	14.7	2.7	11.9	31.6	31.0	18.0	0.8	1.4	2.0	8.1	11.4	5.7
Married (reference)	56.5	80.6	95.5	82.6	59.3	63.8	76.1	97.8	95.4	96.4	86.7	83.6	88.5
Other/Missing	5.8	4.8	1.8	5.6	9.1	5.3	5.9	1.4	3.2	1.6	5.2	5.0	5.9
Parity, %													
Primiparous	43.6	37.0	42.1	38.4	36.8	38.2	38.1	33.3	45.1	34.6	47.2	41.9	44.3
Multiparous (reference)	55.3	62.1	57.7	61.2	63.3	61.3	61.7	66.4	54.7	64.5	52.5	57.6	55.4
Missing	1.1	0.9	0.2	0.5	0.0	0.6	0.3	0.3	0.2	0.8	0.3	0.5	0.3
Province of residence, %													
Québec (reference)	38.0	19.5	88.3	35.8	99.2	45.3	44.5	34.9	14.5	59.6	14.7	28.2	3.3
British Columbia	16.3	32.5	3.3	26.0	0.2	11.4	18.7	23.4	62.8	8.7	51.0	33.9	63.3
Prairies^a^	31.7	33.5	6.7	34.8	0.5	38.7	35.2	39.4	21.5	26.4	32.8	32.6	30.7
Other^b^	14.0	14.5	1.7	3.4	0.1	4.7	1.6	2.3	1.2	5.3	1.6	5.3	2.8
Birth cohort, %													
1990–1993 (reference)	28.9	31.2	17.2	18.0	26.6	29.4	22.4	11.4	19.4	24.0	17.9	30.2	19.3
1994–1997	25.9	26.8	20.6	22.3	27.6	27.6	25.6	18.3	25.5	25.7	27.4	26.2	30.1
1998–2001	22.8	21.9	20.4	25.4	23.7	23.5	23.6	27.8	26.3	23.5	26.4	22.1	26.4
2002–2005	22.3	20.0	41.7	34.3	22.1	19.2	28.4	42.6	28.8	26.8	28.3	21.5	24.2
Total number of births	2,856,394	41,601	17,991	24,339	20,057	10,499	38,702	7,500	68,558	27,491	138,024	108,515	10,970
Share of total births (%)	84.7	1.2	0.5	0.7	0.6	0.3	1.1	0.2	2.0	0.8	4.1	3.2	0.3

Universe consists of singleton live births to women residing in Canada (excluding Ontario) with non-missing information on mother’s birthplace and maternal and infant characteristics

χ^2^ statistic indicates that differences in proportions by maternal birthplace for all covariates are statistically significant at *p* < 0.001 level

^a^Prairie provinces include Alberta, Manitoba, Saskatchewan, and Winnipeg

^b^Includes Atlantic provinces (Prince Edward Island, Newfoundland & Labrador, Nova Scotia, New Brunswick), Territories (Yukon, Northwest Territory, Nunavut), and unknown Canadian province of residence

Neonatal and postneonatal mortality rates by nativity status and birth cohort are depicted in Fig. 1. The general trend is a decrease in infant mortality across successive birth cohorts for all women, regardless of nativity status. However, immigrants consistently had lower death rates than Canadian-born women. Moreover, the nativity gap was relatively stable over time for neonatal mortality (native-immigrant difference of either 0.3 or 0.4 deaths per 1000 live births per cohort). Notably, a statistically significant nativity difference in neonatal mortality was found only for the 1998–2001 cohorts. For postneonatal mortality, the nativity gap was relatively high for the earliest (1990–1993) birth cohorts, with a difference of 0.7 deaths per 1000 live births. But the gap diminished by 57 % over time, resulting in a difference of 0.3 deaths per 1000 live births for the most recent (2002–2005) cohorts. There were statistically significant differences by nativity status across all birth cohorts in the postneonatal period.

Table 2 shows the crude neonatal and postneonatal mortality rates, along with relative and absolute rate differences, by nativity status and maternal birthplace. Canadian-born women serve as the reference group for all comparisons. Overall, the neonatal death rate for immigrants was 16 % lower than the rate for Canadian-born women. Not all immigrant subgroups shared this foreign-born survival advantage, however. Compared to Canadian-born women, neonatal mortality rates for Haitian migrants were 116 % and higher. Non-Spanish Caribbean and Pakistani migrants also had higher crude NMRs than the Canadian-born population but differences were not statistically significant. As a group, immigrants’ survival advantage extended to the postneonatal period as well. Survival during the postneonatal period was especially strong for immigrants from North Africa, Latin America, South Asia, East/Southeast Asia, Europe, and rest of the world, with mortality rates that were 38 % to 53 % *lower* than the Canadian-born rate. In contrast, Pakistani migrants stand out as a subgroup whose offspring are particularly vulnerable to postneonatal mortality, with a rate that is 71 % higher than the one observed for Canadian-born women.

**Table 2:** Crude neonatal and postneonatal mortality rates (per 1,000 live births) for singleton live births by mother’s nativity status and birthplace: Canada (excluding Ontario), 1990–2005

	Neonatal mortality			Postneonatal mortality		
	NMR (95 % CI)	Absolute Difference (rate)	Relative Difference (%)^b^	PNMR (95 % CI)	Absolute Difference (rate)	Relative Difference (%)^b^
Total^a^	2.57 (2.51–2.62)	---	---	1.71 (1.66–1.75)	---	---
Canadian-born	2.63 (2.57–2.69)	Reference	Reference	1.80 (1.75–1.85)	Reference	Reference
Foreign-born	**2.22 (2.09–2.35)**	-0.41	-15.59	**1.20 (1.10–1.29)**	-0.60	-33.33
Among foreign-born						
US	**1.95 (1.52–2.37)**	-0.68	-25.86	1.54 (1.16–1.92)	-0.26	-14.44
North Africa	2.06 (1.39–2.72)	-0.57	-21.67	**0.89 (0.45–1.32)**	-0.91	-50.56
Sub-Saharan Africa	3.04 (2.35–3.73)	0.41	15.59	1.52 (1.03–2.01)	-0.28	-15.56
Haiti	**5.68 (4.64–6.72)**	3.05	115.97	1.79 (1.21–2.38)	-0.01	-0.56
Non-Spanish Caribbean	3.66 (2.51–4.80)	1.03	39.16	1.78 (0.98–2.28)	-0.02	-1.11
Latin America	2.71 (2.19–3.23)	0.08	3.04	**0.85 (0.56–1.14)**	-0.95	-52.78
Pakistan	4.00 (2.57–5.43)	1.37	52.09	**3.07 (1.85–4.32)**	1.27	70.56
South Asia	**2.01 (1.66–2.35)**	-0.62	-23.57	**1.00 (0.75–1.24)**	-0.80	-44.44
West/Central Asia	2.27 (1.75–2.79)	-0.36	-13.69	**1.39 (0.98–1.80)**	-0.41	-22.78
East/Southeast Asia	**1.72 (1.51–1.94)**	-0.91	-34.60	**1.12 (0.91–1.25)**	-0.68	-37.78
Europe	**1.92 (1.66–2.18)**	-0.71	-27.00	**1.12 (0.92–1.31)**	-0.68	-37.78
Rest of the World	**1.39 (0.69–2.09)**	-1.24	-47.15	**0.93 (0.35–1.50)**	-0.87	-48.33

Bold indicates immigrant rates for which 95 % CIs do not overlap with the Canadian-born rate

^a^Total IMR (NMR + PNMR) is slightly different from rate reported for Canada by Public Health Agency of Canada (PHAC, 2008) because of sample exclusion criteria imposed in current analysis

^b^Relative difference calculated by dividing the absolute difference by the Canadian-born rate and multiplying by 100

### Multivariate results

Table 3 shows the adjusted HRs and 95 % CIs from Weibull regression models showing the association between maternal birthplace and neonatal mortality, net of maternal age, marital status, parity, province of residence, birth cohort, and infant sex (non-stratified analysis only). The Weibull shape parameter, *p*, for the full sample and sex-stratified analysis is less than one, indicating a decelerated risk of infant mortality over time as expected. The results from the full sample analysis revealed substantially higher risks of neonatal mortality for the infants of Haitian (HR = 2.29; 95 % CI = 1.90, 2.76), Pakistani (HR = 1.87; 95 % CI = 1.31, 2.68), non-Spanish Caribbean (HR = 1.38; 95 % CI = 1.01, 1.89), and Sub-Saharan African (HR = 1.32; 95 % CI = 1.05, 1.66) migrants than comparable infants of Canadian-born women. The stratified analysis showed modest effect modification by infant sex. Among Haitian and Pakistani migrants, the risk of neonatal mortality was slightly higher for boys than girls but sex differences were not statistically significant as evidenced by overlapping 95 % CIs. Interestingly, the high neonatal mortality risk observed for non-Spanish Caribbean migrants relative to the Canadian-born population was driven entirely by excess death among male infants (HR = 2.01; 95 % CI = 1.42, 2.84). For girls, the daughters of non-Spanish Caribbean and Canadian-born women had similar chances of surviving the neonatal period. Three immigrant subgroups exhibited a clear survival advantage in the neonatal period: US (HR = 0.77; 95 % CI = 0.62, 0.96), East/Southeast Asia (HR = 0.75; 95 % CI = 0.65, 0.85), and Europe (HR = 0.78; 95 % CI = 0.68, 0.90). These immigrant subgroups had 22–25 % lower chances of neonatal mortality than their Canadian-born counterparts. And their survival advantages did not significantly differ for male and female infants.

**Table 3:** Adjusted hazard ratios and 95 % confidence intervals from Weibull regression for the association between maternal birthplace and neonatal mortality, net of covariates, for all infants and stratified by infant sex: Canada (excluding Ontario), 1990–2005

	Neonatal mortality		
	All Infants	Male Infants	Female Infants
	Adjusted HR^a^ (95 % CI)	Adjusted HR^b^ (95 % CI)	Adjusted HR^b^ (95 % CI)
Maternal birthplace:			
Canada	1.00	1.00	1.00
US	**0.77 (0.62, 0.96)**	**0.65 (0.48, 0.90)**	0.94 (0.69, 1.27)
North Africa	0.96 (0.70, 1.33)	0.85 (0.54, 1.34)	1.12 (0.70, 1.78)
Sub-Saharan Africa	**1.32 (1.05, 1.66)**	1.31 (0.97, 1.78)	1.33 (0.94, 1.88)
Haiti	**2.29 (1.90, 2.76)**	**2.10 (1.62, 2.73)**	**2.53 (1.93, 3.32)**
Non-Spanish Caribbean	**1.38 (1.01, 1.89)**	**2.01 (1.42, 2.84)**	0.57 (0.27, 1.19)
Latin America	1.10 (0.91, 1.33)	1.00 (0.76, 1.30)	1.24 (0.94, 1.65)
Pakistan	**1.87 (1.31, 2.68)**	**1.83 (1.13, 2.94)**	**1.93 (1.12, 3.34)**
South Asia	0.96 (0.80, 1.14)	0.92 (0.72, 1.16)	1.02 (0.78, 1.33)
Central/West Asia	1.00 (0.79, 1.26)	0.91 (0.66, 1.26)	1.10 (0.78, 1.55)
East/Southeast Asia	**0.75 (0.65, 0.85)**	**0.70 (0.58, 0.84)**	**0.81 (0.67, 0.98)**
Europe	**0.78 (0.68, 0.90)**	**0.82 (0.68, 0.98)**	**0.74 (0.60, 0.92)**
Rest of the World	0.62 (0.37, 1.02)	0.58 (0.29, 1.15)	0.67 (0.32, 1.40)
*p*	0.27 (0.26, 0.27)	0.26 (0.25, 0.27)	0.27 (0.26, 0.28)
Number of observations	3,370,641	1,729,451	1,641,190

Bold indicates HRs for which 95 % CIs do not overlap 1.00

^a^Model adjusts for maternal age, marital status, parity, providence of residence, birth cohort, and infant sex

^b^Model adjusts for maternal age, marital status, parity, providence of residence, and birth cohort

Table 4 displays the adjusted HRs and 95 % CIs from Cox regression models showing the association between maternal birthplace and postneonatal mortality, net of maternal sociodemographic characteristics. Much of the survival *dis*advantage for non-Spanish Caribbean migrants previously observed in the neonatal period disappeared in the postneonatal period. Only migrants from Pakistan (HR = 2.67; 95 % CI = 1.77, 4.02) and Haiti (HR = 1.41; 95 % CI = 1.02, 1.97) continued to exhibit higher mortality risk compared to the Canadian-born population in the postneonatal period. There was no significant effect modification by infant sex for Haitian and Pakistani migrants. The survival advantages observed for American and East/Southeast Asian neonates did not carry over to the postneonatal period. In fact, the only subgroups that showed a clear survival advantage relative to the Canadian-born population in the postneonatal period were Latin American (HR = 0.57; 95 % CI = 0.50, 0.80) and European (HR = 0.83; 95 % CI = 0.69, 0.99) migrants.

**Table 4:** Adjusted hazard ratios and 95 % confidence intervals from Cox regression for the association between maternal birthplace and postneonatal mortality, net of covariates, for all infants and stratified by infant sex: Canada (excluding Ontario), 1990–2005

	Postneonatal mortality		
	All Infants	Male Infants	Female Infants
	Adjusted HR^a^ (95 % CI)	Adjusted HR^b^ (95 % CI)	Adjusted HR^b^ (95 % CI)
Maternal birthplace:			
Canada	1.00	1.00	1.00
US	0.98 (0.77, 1.26)	1.11 (0.82, 1.51)	0.80 (0.53, 1.22)
North Africa	1.02 (0.62, 1.67)	1.01 (0.53, 1.96)	1.03 (0.49, 2.18)
Sub-Saharan Africa	1.21 (0.88, 1.68)	0.98 (0.61, 1.58)	1.53 (0.98, 2.37)
Haiti	**1.41 (1.02, 1.97)**	1.45 (0.94, 2.24)	1.36 (0.82, 2.27)
Non-Spanish Caribbean	1.09 (0.69, 1.71)	1.40 (0.83, 2.38)	0.67 (0.28, 1.60)
Latin America	**0.57 (0.40, 0.80)**	**0.54 (0.34, 0.85)**	0.61 (0.36, 1.01)
Pakistan	**2.67 (1.77, 4.02)**	**2.46 (1.39, 4.34)**	**2.95 (1.63, 5.35)**
South Asia	0.82 (0.64, 1.06)	0.81 (0.58, 1.12)	0.85 (0.57, 1.25)
Central/West Asia	1.17 (0.87, 1.57)	1.09 (0.72, 1.64)	1.27 (0.82, 1.96)
East/Southeast Asia	0.90 (0.76, 1.06)	0.97 (0.78, 1.19)	0.81 (0.61, 1.06)
Europe	**0.83 (0.69, 0.99)**	**0.68 (0.53, 0.89)**	1.03 (0.80, 1.33)
Rest of the World	0.70 (0.37, 1.30)	0.59 (0.25, 1.43)	0.85 (0.35, 2.04)
Number of observations	3,361,988	1,724,539	1,637,449

Bold indicates HRs for which 95 % CIs do not overlap 1.00

^a^Model adjusted for maternal age, marital status, parity, providence of residence, birth cohort, and infant sex

^b^Model adjusted for maternal age, marital status, parity, providence of residence, and birth cohort

Finally, the Québec subanalysis revealed that group differences in maternal education are insufficient to explain excess neonatal mortality among Haitian migrants (Table 5). For all models, maternal education had the expected effect on neonatal and postneonatal mortality: lower levels of maternal education were associated with higher mortality risk. However, adjustment for maternal education did not attenuate the HRs for Haitian women. The adjusted risk of neonatal death remained twice as high for the offspring of Haitian immigrants as that of Canadian-born women (HR = 2.13; 95 % CI = 1.76, 2.57), with similar effects for girls (HR = 2.37; 95 % CI = 1.80, 3.13) and boys (HR = 1.95; 95 % CI = 1.49, 2.53). Notably, adjusting for maternal education diminished all of the differences in postneonatal mortality risk between Haitian-origin and Canadian-born women (HR = 1.26, 95 % CI: 0.90, 1.76 in Table 5 versus HR = 1.41, 95 % CI = 1.02, 1.97 in Table 4). The findings indicate that group differences in maternal education, while important, may not fully account for maternal birthplace differences in infant death particularly in the early stages of infancy.

**Table 5:** Adjusted hazard ratios and 95 % confidence intervals for the associations between maternal birthplace and education and neonatal/postneonatal mortality, net of covariates, for all infants and stratified by infant sex: Haitian-origin and Canadian-born women in Québec, 1990–2005

	Neonatal mortality^a^	Postneonatal mortality^b^
	Adjusted HR (95 % CI)	Adjusted HR (95 % CI)
All Infants		
Maternal birthplace		
Canada	1.00	1.00
Haiti	**2.13 (1.76, 2.57)**	1.26 (0.90, 1.76)
Maternal education		
Less than high school	**1.45 (1.28, 1.64)**	**1.91 (1.64, 2.24)**
High school	**1.32 (1.16, 1.50)**	**1.54 (1.30, 1.82)**
Some college	**1.13 (1.02, 1.25)**	1.09 (0.94, 1.26)
University or higher	1.00	1.00
Missing	**1.71 (1.51, 1.94)**	**1.58 (1.30, 1.91)**
Number of observations	1,104,063	1,101,264
Male infants		
Maternal birthplace		
Canada	1.00	1.00
Haiti	**1.95 (1.49, 2.53)**	1.30 (0.84, 2.02)
Maternal education		
Less than high school	**1.48 (1.26, 1.75)**	**1.98 (1.61, 2.42)**
High school	**1.40 (1.19, 1.65)**	**1.52 (1.22, 1.89)**
Some college	1.12 (0.98, 1.28)	1.04 (0.86, 1.26)
University or higher	1.00	1.00
Missing	**1.76 (1.49, 2.08)**	**1.63 (1.27, 2.09)**
Number of observations	566,874	565,273
Female infants		
Maternal birthplace		
Canada	1.00	1.00
Haiti	**2.37 (1.80, 3.13)**	1.21 (0.72, 2.03)
Maternal education		
Less than high school	**1.41 (1.17, 1.70)**	**1.84 (1.44, 2.35)**
High school	1.21 (0.99, 1.48)	**1.57 (1.21, 2.03)**
Some college	1.14 (0.98, 1.33)	1.15 (0.92, 1.45)
University or higher	1.00	1.00
Missing	**1.65 (1.36, 2.01)**	**1.51 (1.12, 2.04)**
Number of observations	537,189	535,991

Bold indicates HRs for which 95 % CIs do not overlap 1.00. Sex-stratified models adjusted for birth cohort, maternal age, marital status, parity, and providence of residence. Non-stratified model also adjusted for infant sex along with other covariates in the sex-stratified models

^a^Hazard ratios and 95 % CIs from Weibull model in proportional hazards metric

^b^Hazard ratios and 95 % CIs from Cox proportional hazards model

## Discussion

This study showed that neonatal and postneonatal mortality rates were much lower for immigrants than the Canadian-born population. But there were also important variations, especially in the neonatal period, among immigrant subgroups. In particular, the offspring of Sub-Saharan African, Haitian, non-Spanish Caribbean, and Pakistani migrants had higher risks of neonatal death than comparable children of Canadian-born women, even after taking into account confounding factors. However, in the postneonatal period there were fewer disparities in infant death, with excess mortality observed only among the offspring of Pakistani and Haitian migrants. Sex-stratified analyses revealed little effect modification by infant sex, suggesting that purported son preferences within certain immigrant populations in Canada [42] may not necessarily result in higher postnatal death for girls. Overall, the findings indicate that inequalities in infant mortality are more concentrated in the neonatal period. Contingent on surviving the first 27 days after birth, the infants of most immigrants have the same chances of survival as the infants of Canadian-born women.

The inequalities observed in neonatal mortality may reflect group variation in problems during pregnancy and childbirth. Pregnancy complications such as pre-eclampsia and gestational diabetes increase the chances of preterm labor and prematurity is highly correlated with infant morbidity and mortality [43]. For example, research from Norway showed that the prevalence of pre-eclampsia was lower for Pakistani migrants than Norwegian women. Yet, among women with pre-eclampsia, the preterm birth rate for Pakistani migrants was 42 % higher than that observed for Norwegian women [44]. Unfortunately, comparable population-level estimates of pre-eclampsia rates for Pakistani and other migrant groups are not available in Canada because information about pregnancy and parturition is not available in the birth record data. Thus, it is not possible to discern whether the elevated risk of neonatal mortality among Sub-Saharan African, Haitian, non-Spanish Caribbean, and Pakistani migrants in Canada are the result of a greater distribution of pregnancy and childbirth complications. Prior research in Canada has documented high rates of preterm birth for Haitian migrants, suggesting that possible complications during pregnancy and parturition may be relevant [45]. However, less is known about preterm birth among Pakistani and Sub-Saharan African migrants. Information on gestational age is available on the birth records, thus permitting adjustment for preterm delivery in the linked data. But considering that there may be unobserved factors affecting both preterm birth and infant mortality [46], it is not clear whether adjustment for gestational age would help to further explicate the group disparities in neonatal death or obscure them.

Environmental factors are a major determinant of death in the postneonatal period [47]. For instance, non-biological conditions such as infant sleep position affect the likelihood of sudden infant death syndrome (SIDS), a leading cause of postneonatal death [48]. In Canada, SIDS consistently ranked among the top five leading causes of infant death between 2004 and 2008 [49]. Research on temporal trends in SIDS [50] and SIDS risk by neighborhood socioeconomic conditions [2] has been examined in Canada. But to date, research has not investigated the prevalence of SIDS among specific immigrant subgroups. Future investigation into cause-specific infant mortality by maternal birthplace may shed light on the role of environmental factors in Pakistani and Haitian migrants’ excess postneonatal mortality risk.

Limited access to postpartum care may also contribute to the higher mortality among some of the immigrant subgroups. A longitudinal study of migrant women who delivered in Montreal and Toronto revealed higher rates of postpartum health concerns and unmet health care needs for the infants of immigrant women than comparable infants of Canadian-born women [51]. Barriers related to language or immigrant class (e.g., refugee status) may limit migrants’ ability to access postpartum care for their infants. Further research is needed in order to better understand challenges to adequate postpartum care and its relation to infant mortality, if any, among migrants in Canada.

The overall findings in this study are consistent with the healthy immigrant effect literature on adult mortality in Canada. As a group, adult immigrants in Canada not only have lower mortality than their Canadian-born counterparts [10] but this study demonstrates that they are also able to pass on their survival advantage to their Canadian-born offspring. However, unlike the research on adults where the survival advantage is nearly universal for all migrants irrespective of country/region of origin, there is greater heterogeneity in infant mortality by maternal birthplace. The observed nativity differentials in infant mortality mirror those found in the US [8, 9], where the US-born offspring of immigrants also enjoy a survival advantage. But this foreign-born health advantage is largely absent in Europe where infant mortality rates are typically higher among migrants than the native-born population [52–55]. Future research should explore the role of positive health selection to better understand the maternal birthplace differences in neonatal mortality observed in Canada as well as the cross-national variation in migrants’ infant survival advantage noted here.

This study is not without limitations. First, not all infant deaths over the study period were linked to live birth records. Omission of Ontario data, the province with the most problematic record linkage [17], significantly reduces the proportion of unlinked deaths in the analytical sample. Nonetheless, potential bias stemming from misclassification of births as right-censored remains. It is not possible to determine the magnitude or the direction of the bias because information about the exact number of unlinked deaths per birth cohort and whether the unlinked deaths were to Canadian-born or immigrant women is unknown. Second, it was not possible to identify multiple births to the same woman over the study period. This may have resulted in underestimation of standard errors. It was also not possible to examine the associations between infant mortality and maternal behaviors (e.g., use of prenatal care, alcohol consumption, cigarette smoking, etc.) during pregnancy due to lack of information on the birth certificate. Group differences in maternal behavior during pregnancy may have accounted for some of the variation in infant mortality observed between immigrant subgroups and Canadian-born women. Another important determinant of infant mortality is economic resources (e.g., income) [56]. However, the data did not contain a direct measure of maternal or household income thus limiting the explanatory power of the present analysis to fully account for group differences in neonatal and postneonatal mortality. It was also not possible to examine whether and how immigrants’ duration of residence in Canada affected their chances of infant mortality due to the lack of migration-specific variables on the birth and death records. Research indicates that migrants lose their health advantage the longer they remain in the receiving country [10], and some of the subgroup variation observed in this study may reflect group differences in duration of residence. Finally, the omission of Ontario data limits the generalizability of the findings to all immigrant and non-immigrant populations in Canada. Additionally, it was not possible to access more recent data and changes in the composition of immigrants over time may render the findings less applicable to more recent immigrant cohorts.

## Conclusions

The study provided an analysis of nativity status and maternal country/region of origin differentials in neonatal and postneonatal mortality in Canada. Both neonatal and postneonatal mortality was substantially lower among the offspring of immigrant than Canadian-born women. Exceptionally, migrants from Haiti and Pakistan deviate from this general pattern, with higher mortality rates than the Canadian-born population throughout infancy. Subgroup differences aside, the study also showed that most of migrants’ survival advantages as well as *disadvantages* are concentrated in the neonatal period. Maternal birthplace differences in infant mortality may reflect underlying group differences in biological endowments, human capital, and pre- and post-migration experiences that impact on maternal and infant health [57]. Further population-based and observational research on the perinatal health of Haitian and Pakistani migrants is needed to better understand the biomedical, behavioral, socioeconomic, and health systems risks associated with infant mortality for these subgroups.

## Appendix: Mother’s country/region of origin classification

The 2013 United Nations world region classification [27] was used as a basis for grouping foreign-born mothers into distinct origin countries/regions. Within these broad world regions, countries were also categorized according to similarities in origin cultures. The Spanish-speaking Caribbean countries (Cuba and the Dominican Republic) were grouped into the Latin America category along with Mexico and other Central and South American countries. Guyana and Suriname, despite being geographically located in South America, were grouped into the non-Spanish Caribbean category because these two countries are culturally part of the Anglophone Caribbean [58]. South Asia was separated from the rest of Asia because of the large flows of South Asian migrants in Canada [59]. Additionally, Afghanistan and Iran are considered part of the South Asia region under UN classifications but were grouped with West/Central Asia in the analysis in order to make the “South Asia” category consistent with Canada’s visible minority definition [59]. Sub-Saharan African countries were separated from predominantly Arab North African countries (with the exception of Sudan which is included in the former category). Finally, Oceania and other remaining countries were grouped together as rest of the world.

Two countries – Haiti and Pakistan – were classified as stand-alone categories because preliminary analysis showed them to contribute high numbers of infant deaths within their respective regions. For instance, Haiti was separated from the rest of the non-Spanish Caribbean countries because immigrant women from Haiti have an especially high infant mortality rate that is significantly (*p* < 0.05) different from the rate observed for immigrants from other countries in this region. The IMRs for other countries (e.g., China, El Salvador, India, Lebanon, Mexico, Philippines, Vietnam, and the United Kingdom) were also examined in preliminary analysis to determine internal consistency with their respective regional categories. Results indicated that IMRs for these additional countries were consistent with the regional average. Finally, the US was classified into its own category because it does not easily group with other categories.

### Countries

Haiti, Pakistan, and United States of America (50 states and Washington, D.C.).

### Regions

North Africa: Algeria, Egypt, Libya, Morocco, Tunisia, and Western Sahara.

Sub-Saharan Africa: Africa (no country specified), Angola, Benin, Botswana, Burkina Faso, Burundi, Cameroon, Cape Verde, Central African Republic, Chad, Comoros, Congo (Republic of), Cote d’Ivoire, Democratic Republic of the Congo, Djibouti, Equatorial Guinea, Eritrea, Ethiopia, Gabon, Gambia, Ghana, Guinea, Guinea-Bissau, Kenya, Lesotho, Liberia, Madagascar, Malawi, Mali, Mauritania, Mauritius, Mayotte Islands, Mozambique, Namibia, Niger, Nigeria, Reunion, Rwanda, Seychelles, Saint Helena, Sao Tome and Principe, Senegal, Sierra Leone, Somalia, South Africa, Southern Africa (no country specified), Sudan, Swaziland, Togo, Uganda, United Republic of Tanzania, Zambia, and Zimbabwe.

Non-Spanish Caribbean: Anguilla, Antigua and Barbuda, Aruba, Bahamas, Barbados, Belize, Bermuda, Bonaire, Saint Eustatius and Saba, British Virgin Islands, Caribbean (no country specified), Cayman Islands, Curacao, Dominica, Grenada, Guadalupe, Guyana, Jamaica, Martinique, Montserrat, Netherlands Antilles, Saint-Barthelemy, Saint Kitts and Nevis, Saint Lucia, Saint Martin, Saint Vincent and the Grenadines, Sint Maarten, Suriname, Trinidad and Tobago, Turks and Caicos Islands, and US Virgin Islands.

Latin America: Argentina, Bolivia, Brazil, Central America (no country specified), Chile, Colombia, Costa Rica, Cuba, Dominican Republic, Ecuador, El Salvador, Falkland Islands, French Guinea, Guatemala, Honduras, Mexico, Nicaragua, Panama, Paraguay, Peru, Puerto Rico, South America (no country specified), Uruguay, and Venezuela.

South Asia: Bangladesh, Bhutan, India, Maldives, Nepal, Southern Asia (no country specified), and Sri Lanka.

Central/West Asia: Afghanistan, Armenia, Azerbaijan, Bahrain, Cyprus, Georgia, Iran (Islamic Republic of), Iraq, Israel, Jordan, Kazakhstan, Kuwait, Kyrgyzstan, Lebanon, Occupied Palestinian Territory, Oman, Qatar, Saudi Arabia, Syrian Arab Republic, Tajikistan, Turkey, Turkmenistan, United Arab Emirates, Uzbekistan, and Yemen.

East/Southeast Asia: Asia (no country specified), Brunei, Cambodia, China (including Hong Kong and Macao), Democratic People’s Republic of Korea, Indonesia, Japan, Lao People’s Democratic Republic, Malaysia, Mongolia, Myanmar, Philippines, Republic of Korea, Singapore, Thailand, Timor-Leste, and Vietnam.

Europe: Aland Islands, Albania, Andorra, Austria, Belarus, Belgium, Bosnia and Herzegovina, Bulgaria, Channel Islands, Croatia, Czech Republic, Denmark, Estonia, Europe (no country specified), Faeroe Islands, Finland, France, Germany, Gibraltar, Greece, Guernsey, Holy See (Vatican), Hungary, Iceland, Ireland, Isle of Man, Italy, Jersey, Latvia, Liechtenstein, Lithuania, Luxembourg, Malta, Monaco, Montenegro, Netherlands, Norway, Poland, Portugal, Republic of Moldova, Romania, Russian Federation, San Marino, Sark, Serbia, Slovakia, Slovenia, Spain, Svalbard and Jan Mayen Islands, Sweden, Switzerland, Ukraine, United Kingdom of Great Britain and Northern Ireland, and (The former) Yugoslav Republic of Macedonia.

Rest of the World: Antarctica, American Samoa, At Sea, Australia, Cook Islands, Fiji, French Polynesia, Greenland, Guam, Kiribati, Marshall Islands, Micronesia (Federation States of), Nauru, New Caledonia, New Zealand, Niue, Norfolk Island, Northern Mariana Islands, Oceania (no country specified), Palau, Papua New Guinea, Pitcairn, Saint Pierre and Miquelon, Samoa, Solomon Islands, Tokelau, Tonga, Tuvalu, US territories (no country specified), Vanuatu, Wallis and Futuna Islands, and World (no country specified).

## Abbreviations

IMR: Infant mortality rate
NMR: Neonatal mortality rate
PNMR: Postneonatal mortality rate
US: United States
RDC: Research Data Centre
CI: Confidence interval
HR: Hazard ratio

## Footnotes

1 Reliable estimates of infant mortality rates for Aboriginal peoples in Canada are currently not available at the national level [4].

2 The non-Spanish Caribbean includes countries that were formerly English, French, and Dutch colonies. These countries are typically distinguished from the two Spanish-speaking Caribbean countries, Cuba and the Dominican Republic, in part because of the different languages and cultures but also because of different histories of colonization in the former Spanish colonies [58, 60].

3 In Québec, the *Collège d’enseignement général et professionnel* (or CEGEP) is a network of colleges that provide pre-university and technical programs to high school graduates. Pre-university programs are shorter in duration (2 years) and are meant to prepare students for matriculation into universities. Technical programs (lasting three years) train students for employment but students who take this stream can also matriculate into bachelor programs at universities [61].
